# Chromatin Regulator SRG3 Overexpression Protects against LPS/D-GalN-Induced Sepsis by Increasing IL10-Producing Macrophages and Decreasing IFNγ-Producing NK Cells in the Liver

**DOI:** 10.3390/ijms22063043

**Published:** 2021-03-16

**Authors:** Sung Won Lee, Hyun Jung Park, Jungmin Jeon, Yun Hoo Park, Tae-Cheol Kim, Sung Ho Jeon, Rho Hyun Seong, Luc Van Kaer, Seokmann Hong

**Affiliations:** 1 Department of Integrative Bioscience and Biotechnology, Institute of Anticancer Medicine Development, Sejong University, Seoul 05006, Korea; insiderjjang@gmail.com (S.W.L.); 0402parkhj@gmail.com (H.J.P.); jjm4165@gmail.com (J.J.); dbsgn703@gmail.com (Y.H.P.); mitalus1@gmail.com (T.-C.K.); 2 Department of Life Science and Multidisciplinary Genome Institute, Hallym University, Chuncheon, Gangwon 24252, Korea; sjeon@hallym.ac.kr; 3 School of Biological Sciences, Institute of Molecular Biology and Genetics, Seoul National University, Seoul 08826, Korea; rhseong@snu.ac.kr; 4 Department of Pathology, Microbiology and Immunology, Vanderbilt University School of Medicine, Nashville, TN 37232, USA; luc.van.kaer@vumc.org

**Keywords:** SWItch3-related gene (SRG3), macrophages, lipopolysaccharide (LPS), septic shock

## Abstract

We previously showed that ubiquitous overexpression of the chromatin remodeling factor SWItch3-related gene (SRG3) promotes M2 macrophage differentiation, resulting in anti-inflammatory responses in the experimental autoimmune encephalomyelitis model of multiple sclerosis. Since hepatic macrophages are responsible for sepsis-induced liver injury, we investigated herein the capacity of transgenic SRG3 overexpression (SRG3^β-actin^ mice) to modulate sepsis in mice exposed to lipopolysaccharide (LPS) plus d-galactosamine (d-GalN). Our results demonstrated that ubiquitous SRG3 overexpression significantly protects mice from LPS/d-GalN-induced lethality mediated by hepatic M1 macrophages. These protective effects of SRG3 overexpression correlated with the phenotypic conversion of hepatic macrophages from an M1 toward an M2 phenotype. Furthermore, SRG3^β-actin^ mice had decreased numbers and activation of natural killer (NK) cells but not natural killer T (NKT) cells in the liver during sepsis, indicating that SRG3 overexpression might contribute to cross-talk between NK cells and macrophages in the liver. Finally, we demonstrated that NKT cell-deficient CD1d KO/SRG3^β-actin^ mice are protected from LPS/d-GalN-induced sepsis, indicating that NKT cells are dispensable for SRG3-mediated sepsis suppression. Taken together, our findings provide strong evidence that SRG3 overexpression may serve as a therapeutic approach to control overwhelming inflammatory diseases such as sepsis.

## 1. Introduction

SWItch/sucrose non-fermentable (SWI/SNF) is an ATP-dependent chromatin remodeling complex that regulates gene expression by controlling chromatin structure and DNA accessibility to transcription factors [1]. The SWI3-related gene (SRG3) is a core component of various SWI/SNF complexes [2]. BAF155/SMARCC1, a human homolog of mouse SRG3, interacts with myogenic differentiation factor (MyoD) together with other core subunits of the SWI/SNF chromatin-remodeling complex (e.g., BRG1/BRM-associated factor (BAF) 190a, BAF170, BAF60a, BAF53a, BAF47, and β-actin) in HeLa-MyoD cells [3]. SRG3 plays an essential role in the development and function of adaptive immune cells such as B and T cells [4,5]. Furthermore, we previously showed that SRG3 overexpression can modulate the phenotype of innate immune cells such as dendritic cells (DCs) and macrophages [6,7]. To investigate the effect of constitutive SRG3 expression in all tissues, we previously generated SRG3 transgenic (SRG3^β-actin^) mice using a DNA construct expressing SRG3 complementary DNA (cDNA) under the control of the cytomegalovirus (CMV) immediate enhancer/chicken β-actin promoter [8].

Lipopolysaccharide (LPS), an endotoxin, elicits inflammatory responses through signaling by the TLR4–MD2 complex. LPS stimulates antigen-presenting cells (APCs) such as DCs and macrophages to secrete high levels of interleukin (IL) 12. In turn, APC-derived IL12 is responsible for induction of interferon γ (IFNγ) production from natural killer (NK) and natural killer T (NKT) cells [9,10,11,12,13,14,15]. Unlike LPS derived from Gram-negative bacteria that activates TLR4-expressing cells to induce sepsis, glycolipid antigens from Gram-positive pathogens such as group B *Streptococci* activate NKT cells in a CD1d-dependent manner, ultimately resulting in NKT cell-induced septic shock [16,17].

Macrophages are classified into two groups, classical M1 and alternatively activated M2 macrophages, on the basis of their functional differences. Whereas M1 macrophages produce pro-inflammatory cytokines (e.g., tumor necrosis factor α (TNFα), IL6, and IL12), M2 macrophages secrete anti-inflammatory cytokines (e.g., IL10) [18]. During septic shock, M1 macrophages polarized by IFNγ or LPS participate in inflammatory immune responses [19], whereas M2 macrophages play essential roles in repairing tissue injury caused by sepsis-induced inflammation [20]. Moreover, it has been reported that macrophage polarization plays a vital role in the formation of liver fibrosis and hepatoma. For example, M2-polarized macrophages protect hepatocytes against apoptosis in the process of liver fibrosis [21]. Furthermore, M1-like macrophages polarized by the IL6/STAT3 signaling pathway can inhibit tumor cell growth and survival during hepatocellular carcinoma development [22].

We previously demonstrated that ubiquitous SRG3 overexpression downregulates pro-inflammatory DC activation and promotes anti-inflammatory M2 differentiation, consequently resulting in amelioration of experimental autoimmune encephalomyelitis (EAE) [6]. In this study, we investigated whether β-actin promoter-driven SRG3 overexpression affects the pathogenesis of LPS-induced septic shock. Furthermore, we examined whether SRG3 overexpression can influence the activation of both APCs (DCs and macrophages) and NK receptor-expressing immune cells (NK and NKT cells) during sepsis.

## 2. Results

### 2.1. Ubiquitous Overexpression of the SRG3 Chromatin Remodeling Component Protects Mice against LPS/d-GalN-Induced Septic Shock

**Figure 1:**
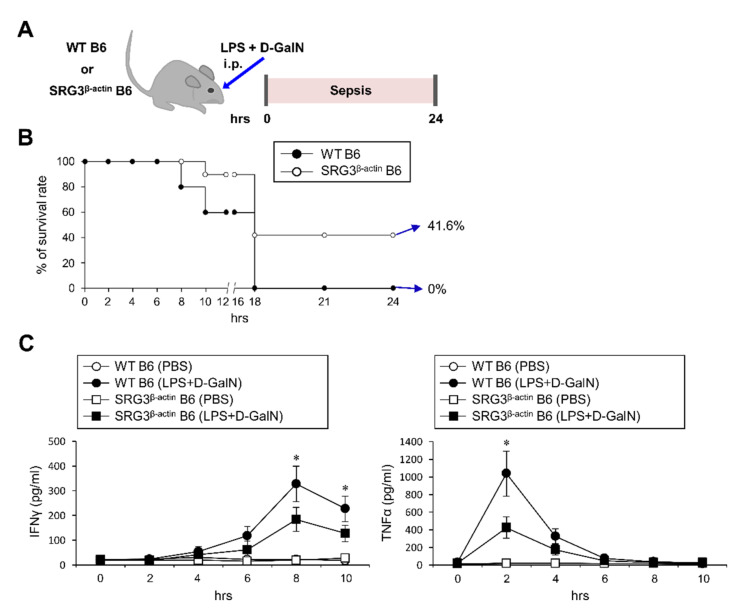
Ubiquitous overexpression of the SWI3-related gene (SRG3) chromatin remodeling component protects mice against lipopolysaccharide (LPS)/ d-galactosamine (d-GalN)-induced septic shock. (**A**–**C**) WT and SRG3^β-actin^ C57BL/6 (B6) mice were intraperitoneally (i.p.) injected with LPS/d-GalN or PBS. (**B**) The survival rates of these mice were monitored until 24 h after LPS/d-GalN challenge (*n* = 7 in WT B6; *n* = 12 in SRG3^β-actin^ B6 mice in the experiment). (**C**) Sera were collected from these mice, and serum levels of interferon γ (IFNγ) and tumor necrosis factor α (TNFα) were measured by ELISA. The mean values ± SD (*n* = 5 per group in the experiment; Student’s *t*-test; * *p* < 0.05) are shown. One representative experiment of two experiments is shown.

We demonstrated that ubiquitous overexpression of SRG3 can modulate EAE development by altering the differentiation of APCs such as macrophages [6]. Here, we examined whether SRG3 overexpression can influence LPS-induced septic shock, which is mediated by IFNγ and TNFα. To test this possibility, we injected wildtype (WT) C57BL/6 (B6) and SRG3^β-actin^ B6 mice intraperitoneally (i.p.) with LPS/2-amino-2-deoxy-d-galactose (d-galactosamine (d-GalN)) (Figure 1A) and assessed the survival rate over a 24 h time period. We found that SRG3^β-actin^ mice treated with LPS/d-GalN displayed significantly reduced mortality rate (58.4%) compared with similarly treated WT mice (100%) (Figure 1B). Moreover, compared with WT control mice, SRG3^β-actin^ mice exhibited reduced levels of serum IFNγ and TNFα (Figure 1C). Therefore, we conclude that ubiquitous overexpression of SRG3 confers resistance to LPS/d-GalN-induced septic shock.

### 2.2. β-Actin Promoter-Driven Overexpression of SRG3 Suppresses LPS/d-GalN-Induced Pro-Inflammatory Cytokine Production in DCs and Macrophages

**Figure 2:**
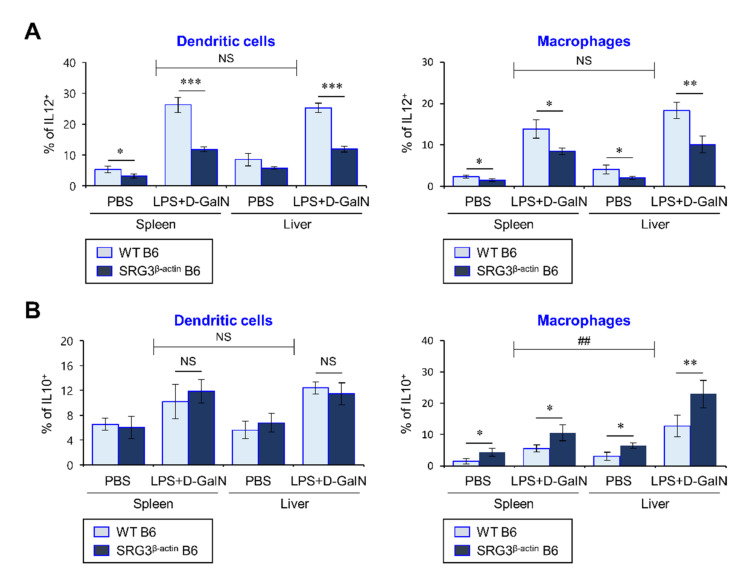
β-Actin promoter-driven overexpression of SRG3 in dendritic cells (DCs) and macrophages suppresses LPS-induced pro-inflammatory cytokine production. (**A**,**B**) WT and SRG3^β-actin^ B6 mice were i.p. injected with LPS/d-GalN or PBS. The spleen and liver were harvested from these mice at 12 h after injection, and the liver MNCs were prepared using a Percoll gradient. (**A**,**B**) The levels of interleukin (IL) 12 (**A**) and IL10 (**B**) in DCs and macrophages were measured by flow cytometry. The mean values ± SD are shown (*n* = 4 per group in the experiment; Student’s *t*-test; * *p* < 0.05, ** *p* < 0.01, *** *p* < 0.001). Two-way ANOVA (tissue × treatment) showed an interaction between these two factors (^##^ *p* < 0.01). One representative experiment of two experiments is shown.

DCs and macrophages can facilitate liver injury during septic shock by secreting pro-inflammatory cytokines such as IL12 and TNFα [23]. Thus, to investigate whether SRG3 overexpression can influence the functional phenotypes (i.e., cytokine production and co-stimulatory molecules) of DCs and macrophages, we injected WT and SRG3^β-actin^ mice i.p. with LPS/d-GalN and measured intracellular levels of IL12 and IL10 in both splenic and hepatic DCs and macrophages. We found that SRG3 overexpression suppresses IL12 secretion by splenic and hepatic DCs but does not affect IL10 production by DCs. Moreover, the M1 (IL12)/M2 (IL10) ratio among macrophages from the spleen and liver was significantly decreased in SRG3^β-actin^ mice compared to WT mice (Figure 2A,B), indicating that SRG3 overexpression polarizes macrophages toward an M2-like phenotype. Intriguingly, increased IL10 production due to SRG3 overexpression appeared to be restricted to macrophages rather than DCs. Thus, our results showed that the inhibitory effects of SRG3 overexpression on the severity of LPS/d-GalN-induced septic shock are associated with a remarkable increase of M2 phenotype macrophages and a significant decrease in the M1 macrophage subset and IL12-producing DCs.

### 2.3. The Inhibitory Effects of SRG3 Overexpression on the Severity of LPS/D-GalN-Induced Sepsis Are Associated with Suppression of NK but Not NKT Cell Activation

**Figure 3:**
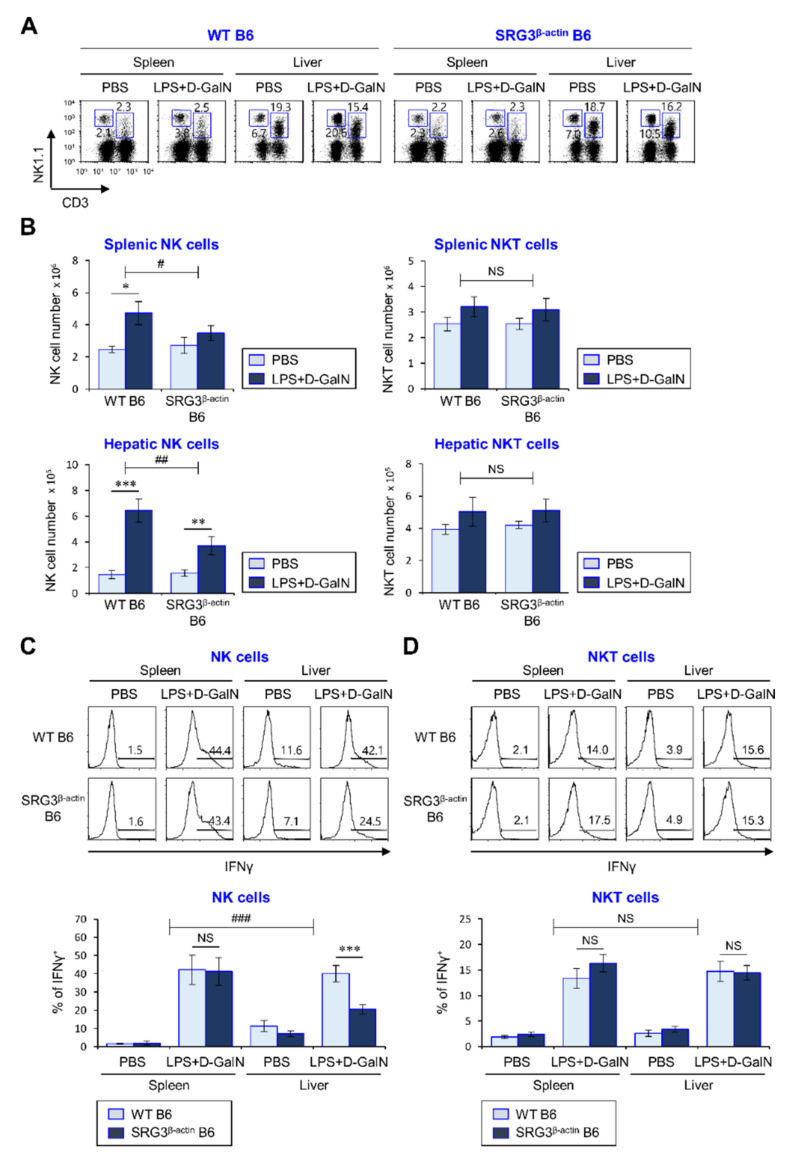
The inhibitory effects of SRG3 overexpression on the severity of LPS/d-GalN-induced sepsis are associated with suppression of natural killer (NK) cell but not natural killer T (NKT) cell activation. (**A**–**D**) The splenic and liver mononuclear cells (MNCs) were prepared as described in the legend to Figure 2. (**A**,**B**) The absolute numbers of splenic and hepatic NK cells and NKT cells from these mice were assessed by flow cytometry at 12 h. Representative data (**A**) and a summary (**B**) are shown. (**C**,**D**) Intracellular IFNγ production in splenic and hepatic NK (**C**) and NKT (**D**) cells was assessed via flow cytometry. The mean values ± SD are shown (*n* = 4 per group in the experiment; Student’s *t*-test; * *p* < 0.05, ** *p* < 0.01, *** *p* < 0.001). Two-way ANOVA (genotype × treatment) showed an interaction between these two factors (^#^ *p* < 0.05, ^##^ *p* < 0.01 and ^###^ *p* < 0.001). One representative experiment of two experiments is shown.

The cytokine IL12 produced by APCs (DCs and macrophages) activates natural killer receptor-expressing innate immune cells (NK cells and NKT cells) to produce pro-inflammatory cytokines (e.g., IFNγ and TNFα) during septic shock, consequently resulting in high lethality [23,24,25]. Since our findings showed that ubiquitous overexpression of SRG3 suppresses inflammatory responses elicited by LPS-activated splenic DCs and macrophages, we evaluated whether the suppressive effect of SRG3 on fatal sepsis is dependent on NK and/or NKT cells. To address this issue, WT and SRG3^β-actin^ mice were i.p. injected with LPS/D-GalN, and the number and activation of NK and NKT cells in the spleen and liver were examined by flow cytometry. We observed remarkable decreases in the number and IFNγ production of NK but not NKT cells in the spleen and liver of SRG3^β-actin^ mice after LPS/d-GalN treatment, compared with WT mice (Figure 3A–D). Moreover, PBS-injected SRG3^β-actin^ mice tended to exhibit decreased IFNγ production by hepatic NK cells compared with PBS-injected WT mice, indicating that the effect of SRG3 overexpression on IFNγ production is likely confined to NK cells rather than NKT cells in the liver. Overall, these results provide strong evidence that NK cells rather than NKT cells play a pivotal role in mitigating severe septic shock mortality.

### 2.4. The Protective Effect of SRG3 Overexpression on LPS/d-GalN-Induced Sepsis Is Independent of NKT Cells

**Figure 4:**
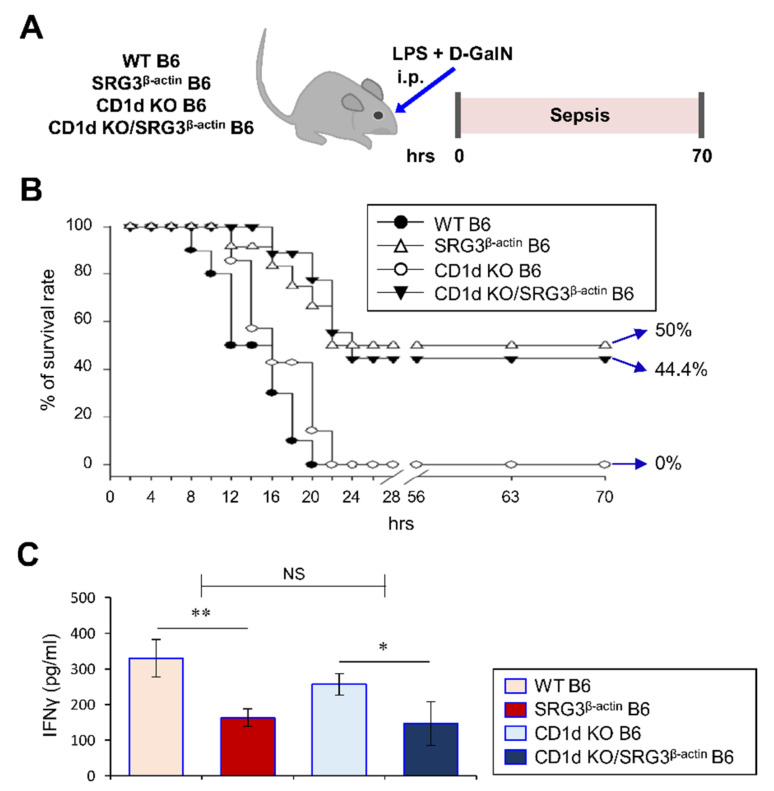
SRG3 overexpression prevents LPS/d-GalN-induced sepsis in an NKT cell-independent manner. (**A**–**C**) WT, SRG3^β-actin^, CD1d KO, and CD1d KO/SRG3^β-actin^ mice were i.p. injected with LPS/d-GalN or PBS. (**B**) The survival rates of these mice were monitored every 2 h starting from LPS/d-GalN injection for a total of 70 h (*n* = 10 in WT B6; *n* = 12 in SRG3^β^^-actin^ B6; *n* = 9 in CD1d KO; *n* = 9 in CD1d KO/SRG3^β-actin^ B6 mice in the experiment). (**C**) Sera were collected from these mice at 10 h after injection, and serum levels of IFNγ were measured by ELISA. The mean values ± SD are shown (*n* = 5 per group in the experiment; Student’s *t*-test; * *p* < 0.05, ** *p* < 0.01). Two-way ANOVA (SRG3^β^^-actin^ × NKT) showed an interaction between these two factors.

Since we found that SRG3^β-actin^ mice show no significant alterations in the infiltration and activation of splenic and hepatic NKT cells compared with WT mice during septic shock, we investigated whether NKT cells participate in the pathogenesis of LPS/d-GalN-induced septic shock in SRG3^β-actin^ mice. To address this question, we generated NKT cell-deficient SRG3^β-actin^ mice by crossing SRG3^β-actin^ mice with CD1d KO mice that lack NKT cells (Supplementary Materials, Figure S1). We then compared the pathological severity of LPS/d-GalN-induced septic shock between SRG3^β-actin^ mice and CD1d KO/SRG3^β-actin^ mice by measuring the survival rate. The survival rates in NKT cell-deficient CD1d KO/SRG3^β-actin^ mice were not significantly different from SRG3^β-actin^ mice (Figure 4A,B). Furthermore, the absence of NKT cells did not significantly affect the suppression of IFNγ production mediated by SRG3 overexpression (Figure 4C). Collectively, these results strongly indicate that NKT cells are dispensable for the regulatory effects of SRG3 overexpression on LPS/d-GalN-induced septic shock.

## 3. Discussion

In this study, we showed that SRG3 overexpression polarizes hepatic macrophages toward an anti-inflammatory M2 phenotype and reduces the pro-inflammatory function of NK cells (i.e., IFNγ production), dampening the cytokine storm induced by LPS/d-GalN-induced sepsis.

A previous study demonstrated that bacterial pathogens can induce macrophage maturation, followed by enhancement (IFNγ production) of NK cell activation via macrophage-derived IL12 [26]. Furthermore, depletion of M2-type Kupffer cells in the liver resulted in increased liver enzyme (AST and ALT) levels, which are indicators of liver damage [27]. During TLR4 agonist-elicited immune responses, Kupffer cell-derived IL12, IL15, and IL18 induce NK cells to produce IFNγ in the liver, whereas Kupffer cell-derived IL10 exerts an inhibitory effect on NK cell activation in the liver [28]. These previous studies indicate that downregulation of NK cell recruitment and activation by SRG3 overexpression could be explained by elevated levels of hepatic macrophage-derived IL10. Although LPS stimulation could activate both NK and NKT cells to secrete cytokine IFNγ through the TLR4 signaling pathway [25], SRG3 overexpression appeared to diminish the activation of NK cells but not NKT cells in LPS-induced sepsis. Such a selective downregulation of NK cell activation is supported by previous studies that NK but not NKT cells are required for liver injury in LPS-, *Pseudomonas aeruginosa* exotoxin A (PEA)-, and cecal ligation and puncture (CLP)-induced sepsis models [29,30,31].

Our prior report demonstrated that macrophages from SRG3^β-actin^ B6 mice compared with WT B6 mice are polarized toward an arginase-1-expressing M2 phenotype after in vitro rIL4 and LPS stimulation [6]. These results are consistent with our current finding of decreased M1/M2 ratio among macrophages in SRG3^β-actin^ mice on LPS/d-GalN-induced septic shock, suggesting the possibility that β-actin promoter-driven SRG3 overexpression can directly polarize macrophages toward an M2 phenotype. Since our study showed that SRG3^β-actin^ B6 mice display a dramatic increase of IL4^+^ basophils and mast cells in the spleen [6], an alternative possibility is that increased M2 polarization observed in SRG3^β-actin^ mice is indirectly caused by increased IL4-producing innate cells such as mast cells [32]. Thus, further studies are needed to clarify the role of SRG3 overexpression on macrophage polarization.

Since messenger RNA (mRNA) expression levels of a particular gene generally correlate with its protein function in the cells, to compare the level of SRG3 expression in diverse cell types, we analyzed BAF155/SMARCC1 expression in various primary cells from healthy human liver and lung tissues using the human protein atlas website. We found that the SRG3 gene is expressed in not only immune cells (T, B, Kupffer cells) but also nonimmune cells (cholangiocytes, club cells, endothelial cells), which is consistent with a previous study [8] (Supplementary Materials, Figure S2). Interestingly, SRG3 mRNA expression by primary cells resident to lung was much higher than in liver, indicating that liver rather than lung is the leading site of inflammation during sepsis.

A previous study attributed the antibacterial effects of T helper type 1 (Th1)-type responses to the induction of macrophage activation, followed by enhancement of NK cell activation via macrophage-derived IL12 [33]. Because macrophages and NK cells from SRG3^β-actin^ mice display a tendency toward decreased inflammatory responses in LPS-induced sepsis, we considered that these cells might contribute to the protection against LPS-induced sepsis mediated by SRG3 overexpression. Since SRG3 overexpression by NK and T cells in SRG3^CD2^ mice had little effect on sepsis development (Supplementary Materials, Figure S3), we speculate that SRG3 overexpression in macrophages from SRG3^β-actin^ mice is critical for suppressing NK cell activation in these mice.

It has been reported that IFNγ treatment can modulate the expression of BAF155/SMARCC1 in human astrocytoma cell lines [34]. NK cells from melanoma patients showed impaired activation of STAT1, a critical molecule in the IFNγ-mediated signaling pathway, after IL2 stimulation [35]. These previous studies suggest that pro-inflammatory cytokines such as IFNγ from NK cells may enhance Th1-type immune responses via inhibiting SRG3 expression. In this regard, it will be interesting to investigate further whether STAT1 activation or inhibition can regulate NK cell activity in an SRG3-associated manner. Moreover, one previous study demonstrated that lung tumor cells overexpressing Brahma-related gene 1 (BRG1), a SWI/SNF subunit, decreased their proliferation when BRG1 inhibitors were orally administered [36], suggesting that SRG3 overexpression can lead to stabilization of BRG1 to favor the growth of tumor cells that prefer an anti-inflammatory environment. Thus, identifying reagents (i.e., small molecules or proteins) specifically targeting the function of SRG3 might provide one more option for sepsis therapy.

In summary, our results show that ubiquitous overexpression of SRG3 suppresses the outcome of LPS/d-GalN-induced sepsis by polarizing hepatic macrophages toward an M2 phenotype and by suppressing NK cell activation. Our recent study demonstrated that genetic engineering of cancer cells via CRISPR/Cas9 gene editing technology improves CD8^+^ T cell-mediated antitumor immunity [37]. Thus, it will be interesting to examine whether therapeutic engineering of SRG3 gene expression can be employed to control sepsis pathogenesis.

## 4. Materials and Methods

### 4.1. Study Design

This study was designed to determine the effect of SRG3 overexpression on LPS-induced sepsis. To address this issue, gene-modified mice including SRG3^β-actin^, CD1d KO, and CD1d KO/SRG3^β-actin^ B6 mice were injected i.p. with LPS/d-GalN. Subsequently, immune cells (liver leukocytes and splenocytes) and serum were harvested and further analyzed by flow cytometry and ELISA. Sejong University Institutional review board approval was obtained before experiments (SJ-20161102, 11-15-2016).

### 4.2. Mice and Reagents

SRG3^β-actin^ B6 and SRG3^CD2^ B6 mice were provided by Dr. Rho Hyun Seong (Seoul National University, Seoul, Korea). CD1d KO mice were provided by Dr. A. Bendelac (University of Chicago, IL, USA). SRG3^β-actin^ B6 mice were further crossed with CD1d KO mice to obtain CD1d KO/SRG3^β-actin^ B6 mice. All mice used in this study were on a B6 genetic background, maintained at Sejong University, and used for experiments at 6–12 weeks of age. Mice were maintained on a 12 h light/12 h dark cycle in a temperature-controlled barrier facility with free access to food and water. Mice were fed a γ-irradiated sterile diet and provided with autoclaved tap water. Age- and sex-matched mice were used for all experiments. The animal experiments were approved by the Institutional Animal Care and Use Committee at Sejong University (SJ-20161102, 11-15-2016). LPS derived from *Escherichia coli* (serotype 0111:B4) was purchased from Sigma-Aldrich (St. Louis, MO, USA).

### 4.3. Induction of Septic Shock

For induction of septic shock, mice were i.p. injected with LPS (2 µg/mouse) plus d-GalN (25 mg/mouse). All animals were continuously monitored for LPS/d-GalN-induced lethality for 24–72 h after challenge.

### 4.4. Genotyping of Tg and KO Mice

The following procedure was performed to verify the presence of the SRG3 transgene. Genomic DNA samples obtained from tail biopsies were used to amplify an 800 bp fragment that was only detectable in SRG3^β-actin^ mice carrying the SRG3 transgene. The following primers were used for genotyping SRG3^β-actin^ mice by PCR: forward, 5′–GAC TAG ACC AAA CAT CTA CCT C–3′; reverse, 5′–GTC AAC TGA GCG ACT GGA TC–3′. This process is consistent with the protocol used in our previous study [6]. To verify the CD1d KO allele, genomic DNA samples from tail biopsies were used to amplify a 280 bp fragment (Neomycin gene) and a 173 bp fragment (CD1d WT allele). The following primers were used for genotyping the neomycin cassette of CD1d KO mice by PCR: forward, 5′–CTT GGG TGG AGA GGC TAT TC–3′; reverse, 5′–AGG TGA GAT GAC AGG AGA TC–3′. The following primers were used for genotyping the CD1d WT allele by PCR: forward, 5′–AAT AGG ATG TAA AAT GAA AAT GTA TCC–3′; reverse, 5′–GGG TCC ATT CCA GAT ACA AA–3′.

### 4.5. Flow Cytometry

The following monoclonal antibodies (mAbs) from BD Biosciences (San Jose, CA, USA) were used: fluorescein isothiocyanate (FITC)- or phycoerythrin (PE)-Cy7-conjugated anti-CD3ε (clone 145-2C11); PE- or allophycocyanin (APC)-conjugated anti-NK1.1 (clone PK-136); FITC- or APC-conjugated anti-CD11c (clone HL3); PE-Cy7-conjugated anti-CD11b (clone M1/70); FITC- or APC-conjugated anti-CD45 (clone PC61); PE-conjugated anti-IL10 (clone JES5-16E3); PE-conjugated anti-IL12p40 (clone C15.6); FITC- or PE-conjugated anti-IgG1 (isotype control) (clone R3-34). The following mAbs from Thermo Fisher Scientific were used: APC-conjugated anti-F4/80 (clone BM8); PE-conjugated anti-IFNγ (clone XMG1.2). To perform surface staining, cells were harvested and washed twice with cold 0.5% BSA-containing PBS (FACS buffer). To block Fc receptors, the cells were incubated with anti-CD16/CD32 mAbs on ice for 10 min and subsequently stained with fluorescently labeled mAbs. Flow cytometric data were acquired using a FACSCalibur flow cytometer (Becton Dickson, San Jose, CA, USA) and analyzed using FlowJo software (version 8.7; Tree Star, Ashland, OR, USA).

### 4.6. Intracellular Cytokine Staining

For intracellular staining, splenocytes were incubated with brefeldin A, an intracellular protein transport inhibitor (10 μg/mL), in RPMI medium (Gibco BRL, Gaithersburg, MD, USA) for 2 h at 37 °C. The cells were stained for cell surface markers, fixed with 1% paraformaldehyde, washed once with cold FACS buffer, and permeabilized with 0.5% saponin. The permeabilized cells were then stained for an additional 30 min at room temperature with the indicated mAbs (PE-conjugated anti-IFNγ, anti-IL4, anti-IL12, anti-IL10, anti-iNOS, anti-Arginase1, or PE-conjugated isotype control rat immunoglobulin G (IgG) mAbs). Fixation and permeabilization were performed using a Foxp3 staining kit (eBioscience, San Diego, CA, USA) with the indicated mAbs (PE-conjugated anti-T-bet, anti-GATA3, or isotype control rat IgG mAbs). More than 5000 cells per sample were acquired using a FACSCalibur, and the data were analyzed using the FlowJo software package (version 8.7; Tree Star, Ashland, OR, USA).

### 4.7. Isolation of Liver Leukocytes

Mice were anesthetized using a combination of ketamine and xylazine at 40 mg/kg and 4 mg/kg, respectively. They were then perfused via the left heart ventricle with cold sterile PBS for 3 min to remove PBMCs from the blood vessels. The liver was removed after perfusion, cut into small pieces by scissors and a scalpel, and digested with DNase I (Promega, Madison, WI, USA; 1 mg/mL) and collagenase type IV (Sigma, St. Louis, MO, USA; 2.5 mg/mL) for 15 min at 37 °C. Subsequently, the digested tissues were dissociated into single-cell suspensions using combination C Tubes and the gentleMACSTM dissociator (Miltenyi Biotech, Bergisch Gladbach, Germany). The digested tissues were filtered using a 70 μm pore cell strainer (BD Falcon, Franklin Lakes, NJ, USA) and the cells were washed once with PBS (10% FBS). Mononuclear cells were collected from the 40/70% Percoll (GE Healthcare, Chicago, IL, USA) interphase after discontinuous Percoll gradient [13]. The total number of mononuclear cells was determined using 0.4% trypan blue (Welgene, Gyeongsan-si, Korea) and a hemocytometer before washing with PBS and antibody staining.

### 4.8. ELISA

The quantity of TNFα and IFNγ in the culture supernatant was determined using a sandwich ELISA according to the manufacturer’s instructions (BD PharMingen, San Jose, CA, USA). The optical density was measured using an Immunoreader (Bio-Tek ELX-800, Winooski, VT, USA).

### 4.9. Data Collection in the Human Protein Atlas

RNA-seq data of BAFF155/SMARCC1 mRNA expression in healthy human liver and lung tissues were re-organized by the Tissue atlas program using data generated by the human protein atlas [38]. Data credit: Human Protein Atlas. Data summary images were obtained from proteinatlas.org, via https://www.proteinatlas.org/ENSG00000173473-SMARCC1/celltype (accessed on 9 March 2021).

### 4.10. Statistical Analysis

Statistical significance was determined using Excel (Microsoft, Redmond, WA, USA). Student’s t-test was performed for the comparison of two groups (* *p* < 0.05, ** *p* < 0.01, and *** *p* < 0.001 were considered significant in the Student’s *t*-test). Two-way ANOVA analysis was carried out using the VassarStats (http://vassarstats.net/anova2u.html) (accessed on 10 June 2020) (^#^ *p* < 0.05, ^##^ *p* < 0.01, and ^###^ *p* < 0.001 were considered to be significant in the two-way ANOVA).

## Data Availability

The data are available from the corresponding author on reasonable request.

## Supplementary Materials

The following are available online at https://www.mdpi.com/1422-0067/22/6/3043/s1.
