# Supplementary material for: Chromatin Regulator SRG3 Overexpression Protects against LPS/D-GalN-Induced Sepsis by Increasing IL10-Producing Macrophages and Decreasing IFNγ-Producing NK Cells in the Liver
Source: Int J Mol Sci. 2021 Mar 16;22(6):3043. doi: 10.3390/ijms22063043 (PMC8002522; doi:10.3390/ijms22063043)
Supplement: Supplementary file 1 [file ijms-22-03043-s001.pdf]

Supplementary Materials to:

Chromatin Regulator SRG3 Overexpression Protects against LPS/D-GalN-induced Sepsis by Increasing IL10-producing Macrophages and Decreasing IFNγ-producing NK cells in the Liver

This document includes:

-Supplementary figures 1-3

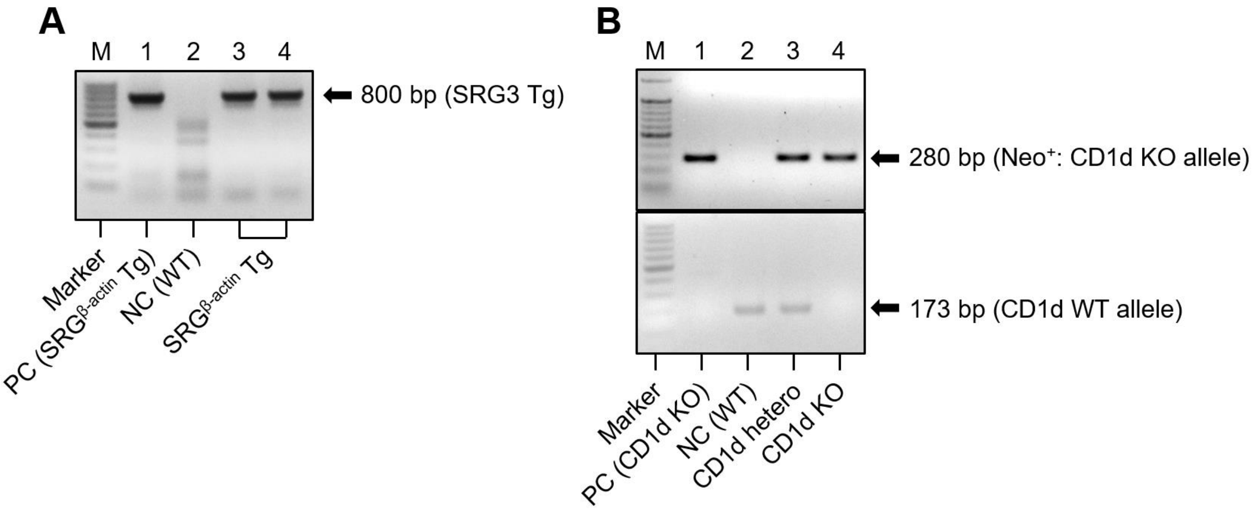

FIGURE S1. Genotyping of the SRG3 Tg and CD1d KO genes.

(**A**) Genotyping of SRG3 Tg gene by PCR analysis. The PCR band size of SRG3 Tg is 800 bp long. M: marker; Lane 1 (+): positive control ($SRG^{\beta\text{-actin}}$ Tg); Lane 2 (−): negative control (wild-type (WT)); Lane 3 & 4: $SRG3^{\beta\text{-actin}}$ Tg. (**B**) Genotyping of CD1d KO gene by PCR analysis. The PCR band sizes of CD1d KO allele (Neo^{+}) and CD1d WT allele are 280 and 173 bp long, respectively. M: marker; Lane 1 (+): positive control (CD1d KO); Lane 2 (−): negative control (WT); Lane 3: CD1d hetero; Lane 4: CD1d KO.

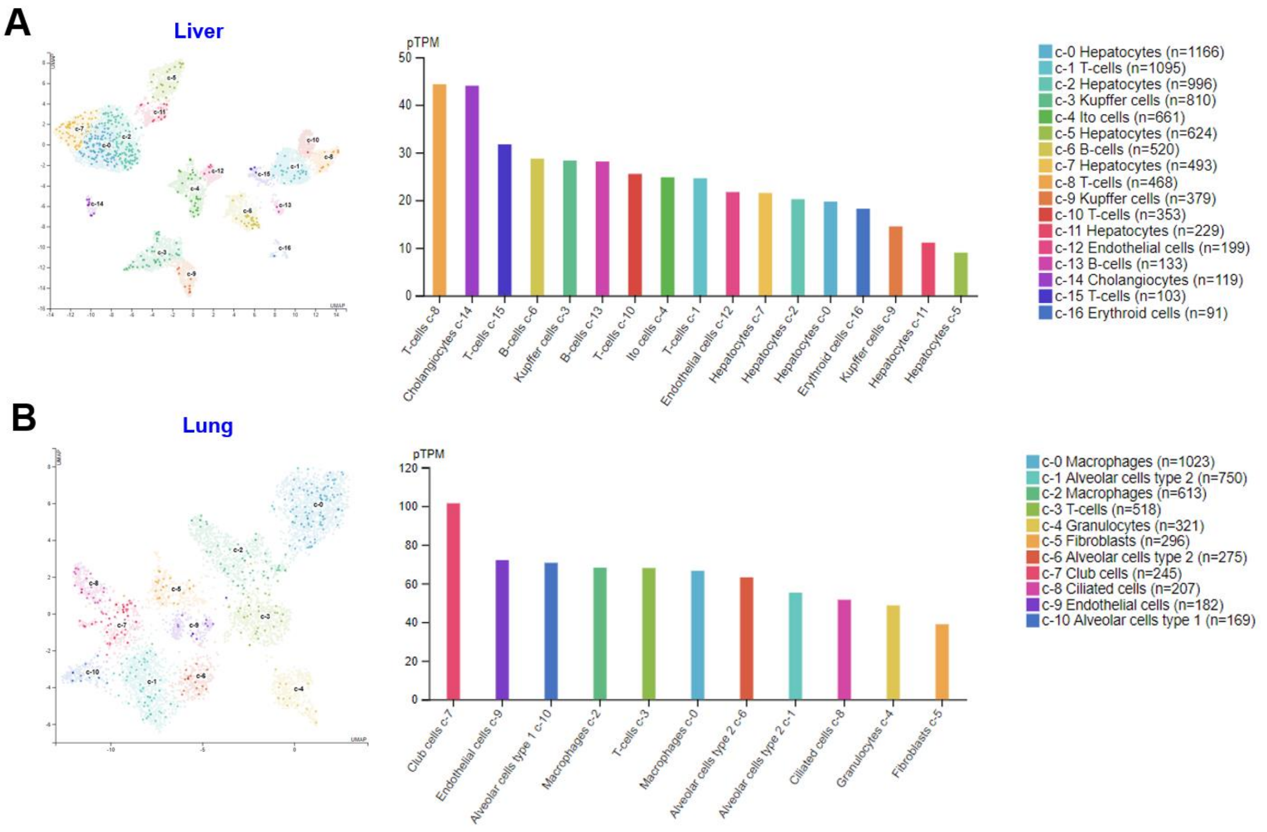

FIGURE S2. Expression pattern of BAF155/SMARCC1 in different cell populations of the human liver and lung.

The mRNA expression profiles of BAF155/SMARCC1 in diverse cell populations of the liver (A) and lung (B) tissues were derived from the human protein atlas (http://www.proteinatlas.org/) and were shown in both scatter and bar plots (pTPM stands for Protein-coding transcripts per million).

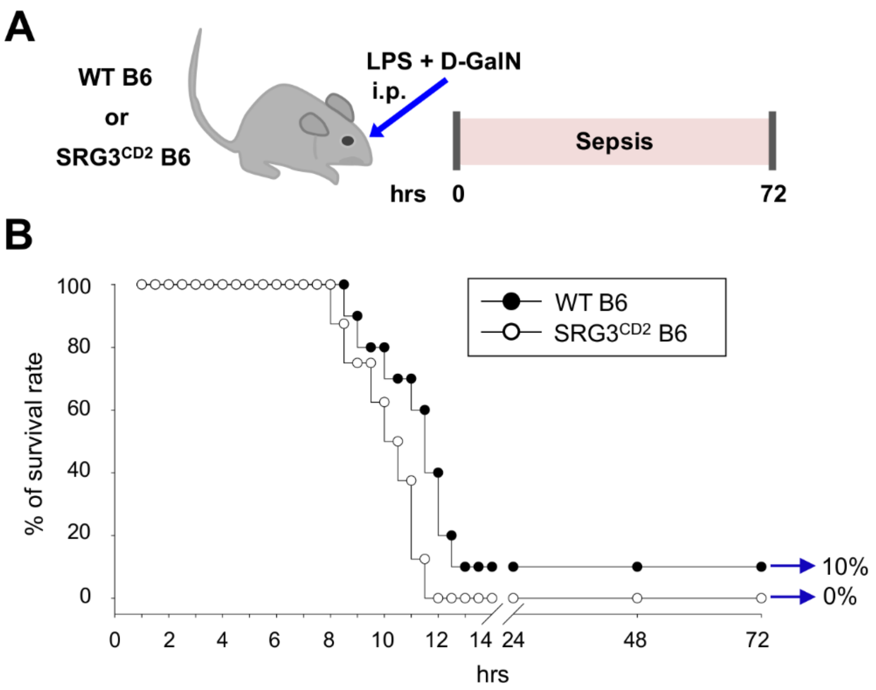

FIGURE S3. SRG3^{CD2} mice develop LPS/D-GalN-induced sepsis similarly to WT mice. (A-B) WT and SRG3^{CD2} mice were i.p. injected with LPS/D-GalN. The survival rates of these mice were monitored every hour, starting from LPS/D-GalN injection for a total of 72 hrs. (n = 10 in WT B6; n = 8 in SRG3^{CD2} B6 in the experiment).
